# Greenhouse gas emissions limited by low nitrogen and carbon availability in natural, restored, and agricultural Oregon seasonal wetlands

**DOI:** 10.7717/peerj.5465

**Published:** 2018-08-28

**Authors:** Laurel Pfeifer-Meister, Laura G. Gayton, Bitty A. Roy, Bart R. Johnson, Scott D. Bridgham

**Affiliations:** 1 Institute of Ecology and Evolution, University of Oregon, Eugene, OR, United States of America; 2 Department of Landscape Architecture, University of Oregon, Eugene, OR, United States of America

**Keywords:** Carbon cycling, Denitrification, Nitrate, Methane, Restoration, Co-limitation, Nitrous oxide, Wet prairies, Mineralization, Pacific Northwest

## Abstract

Wetlands are the major natural source of the greenhouse gas methane (CH_4_) and are also potentially an important source of nitrous oxide (N_2_O), though there is considerable variability among wetland types with some of the greatest uncertainty in freshwater mineral-soil wetlands. In particular, trace gas emissions from seasonal wetlands have been very poorly studied. We measured fluxes of CH_4_, N_2_O, and CO_2_(carbon dioxide), soil nutrients, and net primary productivity over one year in natural, restored, and agricultural seasonal wetland prairies in the Willamette Valley, Oregon, USA. We found zero fluxes for CH_4_ and N_2_O, even during periods of extended waterlogging of the soil. To explore this lack of emissions, we performed a laboratory experiment to examine the controls over these gases. In a fully-factorial design, we amended anaerobic soils from all wetlands with nitrate, glucose, and NaOH (to neutralize pH) and measured production potentials of N_2_, N_2_O, CH_4_, and CO_2_. We found that denitrification and N_2_O production were co-limited by nitrate and carbon, with little difference between the three wetland types. This co-limitation suggests that low soil carbon availability will continue to constrain N_2_O emissions and denitrification in these systems even when receiving relatively high levels of nitrogen inputs. Contrary to the results for N_2_O, the amended wetland soils never produced significant amounts of CH_4_ under any treatment. We hypothesize that high concentrations of alternative electron acceptors exist in these soils so that methanogens are noncompetitive with other microbial groups. As a result, these wetlands do not appear to be a significant source or sink of soil carbon and thus have a near zero climate forcing effect. Future research should focus on determining if this is a generalizable result in other seasonal wetlands.

## Introduction

Wetlands have a significant influence on global climate because of their ability to regulate atmospheric concentrations of the greenhouse gases carbon dioxide (CO_2_), methane (CH_4_) and nitrous oxide (N_2_O), which together comprise 87% of increases in global radiative forcing since 1750 (Myhre et al., 2013). Wetlands are the largest natural source of CH_4_ (Bridgham et al., 2013; Ciais et al., 2013) and a significant source of N_2_O in hydric agricultural soils (Liu & Greaver, 2009), but also can have very high rates of carbon sequestration (Bridgham et al., 2006; Nahlik & Fennessy, 2016). While wetlands provide many important ecosystem services (e.g., flood mitigation, biodiversity conservation, water purification; Millenium Ecosystem Assessment, 2005), this ability to store vast amounts of soil carbon has stimulated interest in establishing wetland carbon offset markets (Galatowitsch, 2009; Hansen, 2009). However, the capacity of wetlands to serve as carbon ‘banks’ is complicated by the potential tradeoff between carbon sequestration and greenhouse gas emissions (Bridgham et al., 2006; Freedman, Stinson & Lacoul, 2009; Neubauer, 2014; Whiting & Chanton, 2001).

Due to their many important services, wetland restoration activities are widespread, both on a voluntary basis and as a legal requirement for mitigating the destruction of natural wetlands in the USA. In newly created or restored wetlands, which are often on former agricultural sites, the potential increase in CH_4_ and N_2_O emissions may offset any gains in soil or aboveground carbon stores, particularly given that the sustained-flux global warming potentials over a 100-yr time frame for CH_4_ and N_2_O are 45 and 270 times greater than CO_2_, respectively (Neubauer & Megonigal, 2015). Thus, the ability of both created and restored wetlands to affect net radiative forcing, either positively or negatively, will in large part depend on the fluxes of these trace gases, the prior state of the restored site, as well as the time period considered (Neubauer, 2014; Whiting & Chanton, 2001). However, little such data exist, with some of the greatest uncertainty (more than 100%) in freshwater mineral-soil wetlands (Bridgham et al., 2013; Bridgham et al., 2006). Because controls on greenhouse gas fluxes vary by wetland type, land use (e.g., disturbed, restored, etc.), seasonality and region, reliable estimates of global and regional emissions will require comprehensive monitoring across ecosystem types (Butterbach-Bahl et al., 2013; Turetsky et al., 2014). Moreover, understanding the controls of these fluxes and how they vary by wetland type will improve our ability to incorporate and parameterize process-based ecosystem models to estimate future emissions under changing climate and land-use (Bridgham et al., 2013).

While many studies have characterized greenhouse gas emissions (particularly CH_4_) in freshwater wetlands (see reviews by Bridgham et al., 2006; Kayranli et al., 2009; Turetsky et al., 2014), far fewer studies have reported values from seasonally inundated wetlands. The vast majority of data from seasonal wetlands come from the prairie pothole region of North America (e.g., Badiou et al., 2011 and references therein); (Tangen, Finocchiaro & Gleason, 2015) or wet prairies of Europe (e.g., Edwards et al., 2014), with only a handful of studies from seasonal wetlands of the US West Coast (Pfeifer-Meister et al., 2012a; Rich & Myrold, 2004), and in no case have the controls of CH_4_ and N_2_O been explored in this system. While relatively undisturbed wet prairies along the interior valleys of the US West Coast now represent a small area, they were once expansive and in most cases have been replaced by farms whose soils continue to remain seasonally wet (Bernert et al., 1999; Duffy, Kahara & Records, 2011; Noss, LaRoe III & Scott, 1995). The West Coast is characterized by a Mediterranean climate, with mild, wet winters and warm, dry summers (National Oceanic and Atmospheric Administration, 1979–2009), which may cause different trace gas dynamics relative to wetlands that receive substantial precipitation during summer months. Many West Coast wetlands also are dominated by 2:1 clays (i.e., Aquerts and Xererts, Soil Survey Staff), which may have considerably different carbon and nutrient dynamics than wetlands with other types of soils (Brady & Weil, 2010; Dinka & Lascano, 2012).

Because of the high historic biological diversity of Pacific Northwest seasonal wetlands, including several federally-listed endemic species (e.g., *Sidalcea nelsoniana*, *Erigeron decumbens var. decumbens*, *Icaricia icarioides fenderi*) (US Fish and Wildlife Service, 2016), the primary goal of wetland prairie restorations in this region is generally to maximize native plant cover and diversity, and associated native fauna, with much less focus on belowground functioning (Pfeifer-Meister et al., 2012a). In particular, no study has examined the rates and the controls over greenhouse gas fluxes in these seasonal wetlands. Thus, our initial objective was to determine how a variety of restoration techniques influence greenhouse gas emissions (CO_2_, CH_4_, and N_2_O) by comparing the restorations to a native, remnant wetland and an adjacent unrestored agricultural field (the prior condition of the restorations). We hypothesized that (1) N_2_O emissions would decrease with restoration due to the cessation of nitrogen fertilization, and (2) minimal changes in CH_4_ flux would be observed between the agricultural field and the restorations due to the intact hydrology of the agricultural field. However, we found zero fluxes of CH_4_ and N_2_O and thus a second objective was derived to examine controls over these gases with a manipulative laboratory experiment. Specifically, we hypothesized that CH_4_ and N_2_O production were inhibited due to low soil carbon and nitrogen availability, respectively, likely reflecting the prolonged dry state of the soils in the summer and the strong sorption capacity of the dominant 2:1 clays found in Vertisols of this region. We also examined if soil acidity was a limiting factor for the production of these gases.

## Methods

### Site descriptions

Wetland prairies in the West Coast of the USA experience a Mediterranean climate, with >90% of the annual precipitation falling between November and May in the Willamette Valley, Oregon (National Oceanic and Atmospheric Administration, 1979–2009). Due in part to the asynchrony between temperature and rainfall, seasonal wet prairies are the most extensive wetland type in the region and historically occupied 10% of the Willamette Valley (Christy & Alverson, 2011). These wetlands are intermittently flooded during the winter months, with a perched water table (∼5–10 cm standing water) on 2:1 shrink swell clays, and completely dry up during the warm summer months (Marshall, 2011). Peak flowering is in mid-June, with almost complete plant senescence by mid-July. Despite their historical dominance, wetland prairies in Oregon’s Willamette Valley are considered a critically endangered ecosystem (Noss, LaRoe III & Scott, 1995) due to severe losses, with an estimated <2% of native wet prairies remaining (Christy & Alverson, 2011; Christy et al., 2011). Similar to wetlands across the US (Dahl, 2011), these losses are largely attributable to agricultural activities and urbanization (Hulse, Baker & Gregory, 2002; Morlan et al., 2010). Despite national and state policies of no-net-loss, wetland area continues to decline in the Willamette Valley (Morlan et al., 2010). Given their imperiled status, wetland prairies are the focus of extensive restoration efforts and much of the potentially restorable wetland area is currently in agricultural production with relatively intact hydrology.

For the field experiment, we measured seasonal gas fluxes and associated soil nutrients in a 4.5 ha experimental restoration site, Coyote Prairie (44°02′26″N, 123°14′43″W) located near Eugene, OR, USA. The soil at this site is in the Natroy series (Very-fine, smectitic, mesic Xeric Endoaquerts) (Soil Survey Staff, 2017). This site was used in the production of *Lolium multiflorum* Lam. (annual ryegrass) seed for 25 years prior to being restored in 2004, but was never drained—a common agricultural practice in the Willamette Valley. During active seed production, the site was burned and tilled annually and fertilized twice annually with 4 g N m^−2^, 2 g P m^−2^, and 3.5 g K m^−2^ (Pfeifer-Meister et al., 2012b). In 2004, part of the site was removed from agricultural production, and large replicate plots (15 m by 15 m, *n* = 5) were restored using 10 different combinations of site preparation techniques. Treatments included various combinations of summer and fall herbicide applications (Glyphomate 41, salt formulation), tilling (field disk and cultipacker to 20 cm depth), thermal (Sunburst infrared burner, Eugene, OR; temperature output 650–800 °C), and solarization (plots covered with 0.15 mm clear plastic for four months). Following treatment implementation all plots were seeded with an identical mix of 15 native species in October 2004. The summer herbicide application had no detectable effect on any below or aboveground response variable measured (*p* > 0.30; likely because the application occurred after plants had senesced); thus we lumped this treatment with its equivalent counterpart, reducing the number of restoration treatments from 10 to seven (Table S1). For a more detailed description of this site and treatments, see Pfeifer-Meister et al. (2012b). In addition to the 50 restoration plots, five plots were sampled in the adjacent agricultural field, which continued to be actively farmed (i.e., tilled, fertilized, burned, harvested). These plots were immediately adjacent to the restoration at the same site (Coyote Prairie) and were randomly located along the entire edge of the experimental restorations.

Because we found zero fluxes of CH_4_ and N_2_O in our seasonal sampling (see ‘Results’) or in a one-time survey of nine other wetland prairies in the region (Pfeifer-Meister et al., 2012a), we conducted a fully factorial laboratory experiment that amended soils with nitrate, carbon, and sodium hydroxide (to increase pH) to determine factors limiting N_2_O and CH_4_ production in these wetland prairies. We were also interested in comparing our experimental restoration to a natural wetland prairie. Thus, for the laboratory experiment, we collected soils from a nearby high-quality remnant, Oxbow West (44°03′26″N, 123°11′29″W) that was included in the one-time survey (Pfeifer-Meister et al., 2012a). This site was never drained or plowed, but is managed with prescribed burns and invasive species removal to maintain native biodiversity. The soil at Oxbow West is in the Dayton series (Fine, smectitic, mesic Vertic Albaqualfs) (Soil Survey Staff, 2017). Because we only collected soils from a single restoration, agricultural, and remnant wetland for the laboratory experiment, land-use state is pseudo-replicated and our ability to extrapolate to other remnants, agricultural fields, and restorations is limited.

### Field experiment

We measured in situ fluxes of N_2_O, CH_4_, and CO_2_ in the second year after restoration (*n* = 50) and the agricultural field (*n* = 5) in the fall (14 October 2005), winter (13 January 2006), and spring (7 April 2006). Fluxes were measured with PVC chambers (10.16 cm diameter, 35 cm tall) placed 5 cm in the ground the previous day after all aboveground plant matter was clipped from the chamber location; hence the CO_2_ flux represents soil respiration only (i.e., plant roots, rhizosphere, soil microbes and fauna). Chambers were sealed with a rubber cap, and headspace samples (20 cm^3^) were collected six times over 2 h and stored in pre-vacuumed serum bottles. Soil temperature was measured at a 5-cm depth adjacent to each chamber. In the summer (5 July 2006), it was impossible in these dry shrink-swell clay soils to insert gas-tight chambers. Therefore, intact soil cores were placed in Mason jars fitted with septa, immediately brought back to the laboratory and incubated in the dark at the average in situ soil temperature (17.5 °C). Gas samples were collected every 30 min over 2 h the same day. CO_2_ and CH_4_ were measured using a FID detector with a methanizer and N_2_O was measured with an ECD detector on a SRI model 8610C gas chromatograph (Torrance, CA, USA) within one week of sample collection. We were capable of measuring sub-atmospheric concentrations of both N_2_O and CH_4_. Flux rates were determined from the linear change in gas concentration over time, and nonsignificant slopes were assigned a flux of zero.

In the fall, winter, and spring after chambers were uncapped, we collected a soil core (5.7 cm diameter, 8.5 cm depth) inside the chamber footprint. In the summer, the soil cores collected for the gas flux measurements were used for additional analyses. In all seasons, an adjacent core was also placed in a Ziploc bag, buried back in its hole, and left in the ground for two weeks to determine net nitrogen mineralization and net nitrification (Hart et al., 1994). We extracted root-free, sub-samples of soil from each core for NO_2_^−^ + NO_3_^−^ and NH_4_^+^ using 2 M KCl (Maynard & Kalra, 1993) and PO_4_^3−^ using 0.5 M NaHCO_3_ (Kuo, 1996) within one day of sample collection. Extracts were frozen until analysis. NO_2_^−^ + NO_3_^−^, NH_4_^+^, and PO_4_^3−^ were measured colorimetrically with an Astoria II autoanalyzer (Astoria Pacific International, Clackamas, OR, USA) using the cadmium reduction (Wood, Armstrong & Richards, 1967), phenate (Solorzano, 1969), and ascorbic acid (Murphy & Riley, 1962) methods, respectively. Microbial biomass carbon and nitrogen were determined using the chloroform-fumigation method (Horwath & Paul, 1994; Voroney & Winter, 1993), and is described in detail in Pfeifer-Meister et al. (2012a).

We measured pH using a 1:1 soil-deionized water slurry by weight. Bulk density was determined by weighing each core after collection and correcting for percent moisture by drying a sub-sample at 60 °C for 48 h. We converted soil moisture to water-filled pore space (WFPS) for each season using bulk density and an average particle density of 2.65 g cm^−3^. We determined soil texture once on soils collected in the fall of 2005 (Gee & Bauder, 1986) and total carbon and nitrogen once on dried, ground soils collected in the summer of 2006 using a Costech Analytical Technologies 4010 elemental combustion analyzer (Valencia, CA, USA). Aboveground net primary productivity (NPP) was estimated in 2006 at peak standing biomass in each of the experimental and agricultural field plots. Within each plot, three 10 cm by 10 cm plots were randomly located and clipped between 20–27 June 2006. Biomass was sorted into graminoids, forbs, and woody plant material, dried at 60 °C for 48 h, and weighed. Biomass was collected in an identical manner at the same time of year in 2005 in the reference plots and is included for comparison (Pfeifer-Meister et al., 2012a).

### Laboratory experiment

To determine factors limiting N_2_O and CH_4_ production in these prairies, we conducted a fully factorial laboratory experiment that amended soils with nitrogen as KNO_3_, carbon (C) as glucose, and NaOH to adjust the pH to 7.0, along with all factorial combinations of these treatments (i.e., C + N, C + pH, N + pH, and C + N + pH). We additionally had an unamended control (1:1 soil deionized water slurry) and a salt control to determine if the KNO_3_ was having an unintended effect by changing salinity. All soil amendments were replicated five times in each of the three wetland types (restored, agriculture, reference; total *n* = 150).

For this experiment, we collected cores (5.7-cm diameter, 8.5-cm depth) on 30 May 2006 from only the five till + solarization plots (one of the restoration treatments) at Coyote prairie, the five agricultural plots, and five randomly located plots at the remnant wetland prairie. In each of the 15 plots, three soil cores were collected for a total of 15 cores per site. All cores from a site were homogenized and large roots and live plant matter were removed on the following day. We measured NO_2_^−^ + NO_3_^−^ and NH_4_^+^ availability, total carbon and nitrogen, pH and soil moisture on five soil subsamples as described above to determine initial conditions of our three sites.

One day after sample collection approximately 30 g of homogenized, root-free soil was added to a 120-mL serum bottle. All bottles were slurried so that final moisture content was a 1:1 weight of dry soil to deionized water. For the nitrogen amendment, we added 10 mL of a 0.064 M solution of KNO_3_. This amount was 10 times higher than the highest recorded inorganic nitrogen level during the previous year (in the agricultural field during the fall sampling). In the salt treatment, KCl was added at the same molarity as the KNO_3_ treatment. Soils were amended with carbon by adding 10 mL of a 0.028 M glucose solution. This addition was four times maximum carbon respiration rates observed in the spring and fall (multiplied by the number of days for the experiment, including the pre-incubation period). pH was adjusted to 7.0 using a 10% NaOH solution. In cases where the carbon or nitrogen treatment altered pH, the pH was returned to its original value by means of 10% NaOH. Bottles were capped with grey butyl septa, crimped, and flushed with N_2_ for 20 min to ensure soils were anaerobic. The bottles were pre-incubated at 19 °C for five days.

After the five day pre-incubation period, the samples were flushed again with N_2_ for 20 min. A 20-mL sample was taken at time zero and after 24 h to determine CO_2_, CH_4_, and N_2_O production. All sampled gas was replaced with nitrogen and accounted for with dilution factors. After 24 h, denitrification was measured by replacing approximately 10% of the headspace with CaC_2_-generated acetylene (C_2_H_2_) (Drury et al., 2008). We collected two final gas samples 5 and 24 h after the C_2_H_2_ addition (hours 29 and 48 of the incubation). Acetylene inhibition has been demonstrated to systematically underestimate rates of denitrification (Butterbach-Bahl et al., 2013), but we were interested in mechanistically determining the limits on denitrification and did not attempt to ascribe these laboratory measurements to in situ rates. Additionally, the N_2_O assay was not dependent on acetylene inhibition, and the high correlation between the N_2_O and denitrification rates (see ‘Results’) suggests that the latter were not particularly biased in any way among the treatments.

### Statistical analyses

For the field experiment, we performed one-way ANOVAs and used Tukey’s post hoc pairwise comparisons to examine the effect of restoration treatment on bulk density, total carbon, total nitrogen, soil texture (% clay, % silt, % sand), and aboveground NPP (*n* = 55). We used repeated-measures ANOVAs to determine the effect of restoration treatment and season on greenhouse gas production, nutrient concentrations (NH_4_^+^, NO_2_^−^ + NO_3_^−^, and PO_4_^3−^), net nitrogen mineralization, net nitrification, microbial biomass (carbon and nitrogen), % moisture, and pH (*n* = 220). When season significantly interacted with restoration treatment, we compared treatments within a season using Tukey’s post hoc tests. If there was no interaction, we examined differences in treatments averaged across all seasons using Tukey tests. Differences among seasons were examined using pairwise comparisons. Response variables were transformed as appropriate to achieve approximate normal distributions (soil respiration, nitrate availability, and the C/N ratio were square root transformed and NH_4_^+^ availability was log transformed) and Greenhouse-Geisser values are reported for the repeated-measures ANOVAs to correct for violations of sphericity.

For the laboratory experiment, we used a 4-way ANOVA to test for differences among our soil amendment treatments and sites (fixed main effects included: site, nitrogen addition, carbon addition, and pH alteration). One bottle was not gas tight and thus was discarded from all analyses (final *n* = 119). To determine if changing salinity had an effect on greenhouse gas production, a one-way ANOVA was conducted comparing the control treatment with the salinity treatment (*n* = 30). Response variables were transformed as appropriate to meet the assumption of normality (see Table S4 for individual transformations) and Tukey’s post hoc comparisons were used to examine differences among sites and treatments. All statistics were run using SPSS vs. 22 (IBM software, http://www-01.ibm.com/software/analytics/spss/). Means ± one standard error are reported in the results.

## Results

### Field experiment

In all seasons and plots, the headspace concentrations of N_2_O and CH_4_ remained at atmospheric concentrations over the course of the two-hour incubations (this could not be attributed to leaky chambers as the *r*^2^’s for CO_2_ flux were ≥0.89 and averaged 0.99). Many of the other measured soil properties, including bulk density, total carbon and nitrogen, carbon/nitrogen ratio, soil texture, phosphate availability, and gravimetric percent moisture, showed no effect of restoration treatment (Table S2). Not surprisingly, season significantly affected all soil response variables measured (*p* < 0.001, see Table S3 for seasonal means of moisture, temperature, and pH).

**Figure 1:**
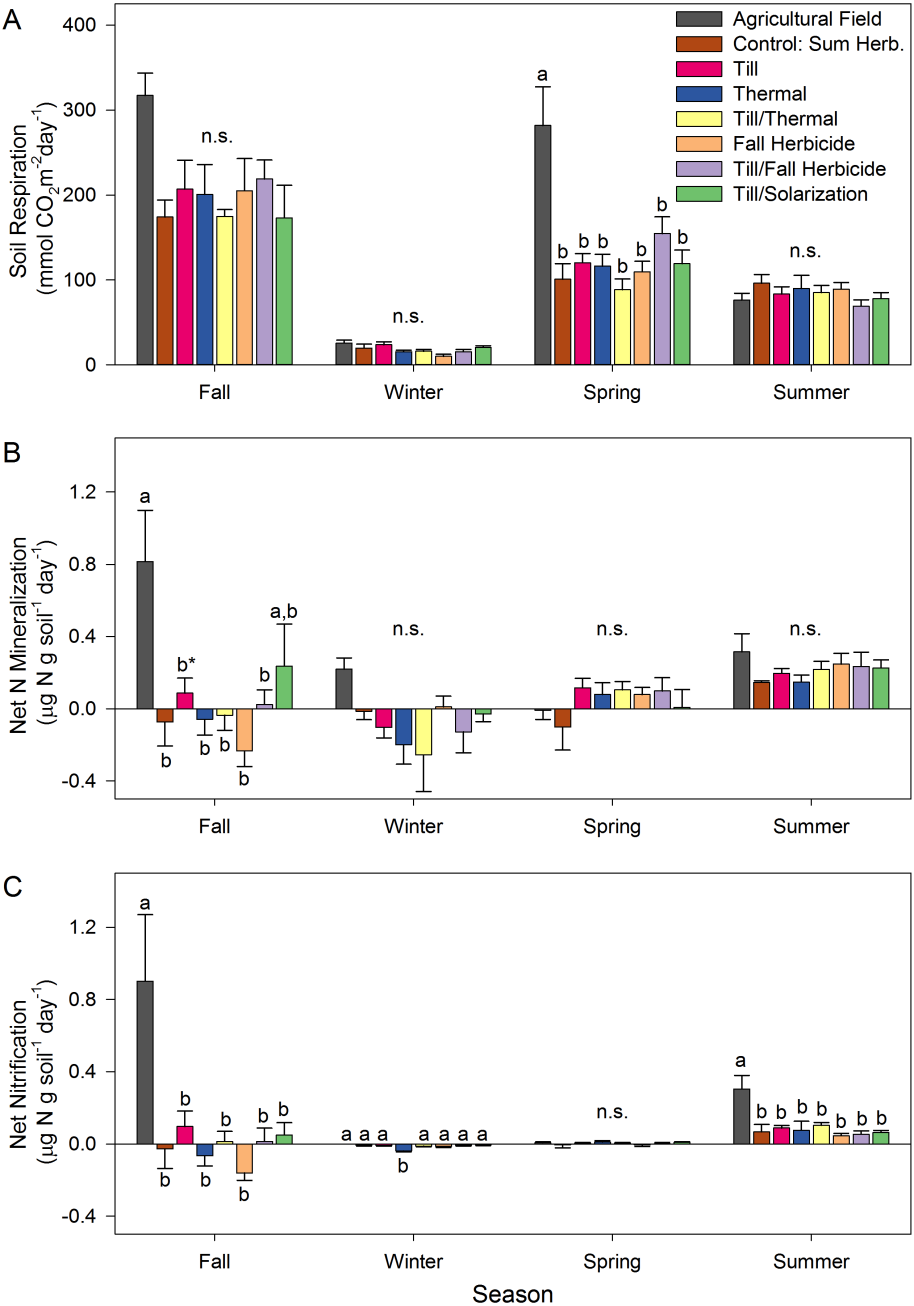
Seasonal soil respiration and nitrogen cycling in restoration treatments and agricultural field. (A) Soil respiration, (B) net nitrogen (N) mineralization, and (C) net nitrification in the fall 2005, winter 2006, spring 2006, and summer 2006 in the agricultural field and restoration treatments. Error bars represent one standard error from the mean and lower case letter differences indicate significant (*p* < 0.05, ^∗^*p* < 0.10) effects of treatment within a season.

Soil CO_2_ flux varied by an order of magnitude across seasons (*p* ≤ 0.001 for all pairwise comparisons), with the highest rates observed in the fall (202 mmol CO_2_ m^−2^ day^−1^ ± 12), followed by the spring (129 ± 9), summer (83 ± 3), and winter (19 ± 1). Restoration treatments only affected soil CO_2_ flux in the spring, when it was approximately three times higher in the agricultural field than in all other restoration treatments (Fig. 1A). Net nitrogen mineralization also varied by season, with summer (0.22 µg N g^−1^ soil day^−1^± 0.02) having higher rates of mineralization than spring (0.05 ± 0.03) or winter (net immobilization, −0.08 ± 0.03, *p* < 0.001, Fig. 1B). Due to high variability, treatment effects were muted, with the only significant response observed in the fall. In this season, net mineralization rates were at least four times greater in the agricultural field than all restoration treatments except the till + solarization treatment (although not significant, this trend remained in both winter and summer). Similarly, net nitrification rates were three to five times higher in the agricultural field than all treatments in the fall and summer (*p* ≤ 0.023, Fig. 1C). In the winter, the thermal treatment had more nitrate immobilization than all other treatments (*p* ≤ 0.037), but rates were very low (ranged from −0.04 µg N g^−1^ soil day^−1^ in the thermal treatment to 0.00 µg N g^−1^ soil day^−1^ in the agricultural field). In the spring, no significant differences were detected for net nitrification.

In the fall, both ammonium and nitrate levels were higher in the agricultural field than in the experimental restoration plots (NH_4_^+^ *p* ≤ 0.042, NO_2_^−^ + NO_3_^−^ *p* < 0.001, Fig. S1). In addition, nitrate availability was lower in the till + solarization treatment than in all other experimental treatments except the till + fall herbicide treatment (Fig. S1B , *p* ≤ 0.082). In the winter, nitrate availability was higher in the thermal treatment than in the control, till, till + fall herbicide, till + solarization, and agricultural field (*p* ≤ 0.029). In the spring, nitrate availability was very low and no differences were detected among treatments, but in the summer the agricultural field had higher nitrate availability than all other treatments and the till + solarization treatment had lower nitrate availability than all other treatments (*p* ≤ 0.005). Ammonium levels did not vary significantly among treatments in the winter, spring, or summer. Available phosphate never varied by treatment (*p* = 0.70, Fig. S1C ), but fluctuated throughout the year with the highest availability in the spring (20.2 µg P g^−1^ soil ± 0.8, *p* < 0.001) and lowest availability in the summer (6.1 µg P g^−1^ soil ± 0.6, *p* < 0.001).

Microbial carbon and nitrogen biomass as well as pH differed significantly among treatments (*p* ≤ 0.005, Fig. S2), and these effects were not dependent upon season (*p* ≥ 0.32, Table S2). The till + solarization treatment had significantly lower microbial carbon than the agricultural field and all treatments other than the till + thermal treatment. Similarly, microbial nitrogen biomass was lower in the till + solarization treatment than all treatments except the control. Finally, the agricultural field was slightly more acidic (4.89 ± 0.04) than the till + fall herbicide, till + solarization, and till + thermal treatments (5.09 ± 0.03, *p* ≤ 0.038).

Aboveground NPP was more than three times higher in the agricultural field than in the experimental restoration treatments and reference wetland (*p* < 0.001, Table S2 & Fig. S3). Though the restoration treatments did not differ in total aboveground NPP, the till + solarization treatment had higher graminoid NPP than the control or till-only treatments and lower forb NPP than all experimental treatments except the fall herbicide and till/fall herbicide treatments.

### Laboratory experiment

The three soils used in the laboratory experiment to examine controls of trace gas production varied in initial soil physical and chemical properties (Table 1). The agricultural field had higher nitrate and ammonium availability than the restored and reference wetlands. The agricultural field and restored wetland had similar bulk density, pH, and soil texture, but were less compact, more acidic, and had a higher clay and lower sand content than the reference wetland. No differences were observed among sites for total carbon and total nitrogen.

**Table 1:** Description of soils used in the laboratory incubations. Pre-treatment description of soils (means  ± 1 SE) used for the laboratory experiment.

	Bulk density (g cm^−3^)	Total C (mg C cm^−3^)	Total N (mg N cm^−3^)	pH	% Clay	% Sand	% Silt	NO${}_{3}^{-}$ (µg N g^−1^ soil)	NH${}_{4}^{+}$ (µg N g^−1^ soil)
Agricultural field	1.10 ± 0.03b	27.3 ± 1.6a	2.2 ± 0.1a	5.0 ± 0.04b	40.3 ± 1.8a	23.4 ± 2.0b	36.3 ± 1.3a	2.5 ± 0.1a	7.4 ± 0.9a
Restored wetland	1.16 ± 0.01b	26.7 ± 1.6a	2.1 ± 0.1a	5.0 ± 0.01b	37.3 ± 1.8a	25.3 ± 2.0b	37.4 ± 1.3a	0.2 ± 0.1b	1.5 ± 0.9b
Reference wetland	1.30 ± 0.03a	23.0 ± 1.6a	2.0 ± 0.1a	5.6 ± 0.04a	26.7 ± 1.8b	33.6 ± 2.0a	39.7 ± 1.3a	0.2 ± 0.1b	5.3 ± 0.9b

**Notes.**

Small letter differences indicate significant differences among sites (*p* < 0.05).

**Figure 2:**
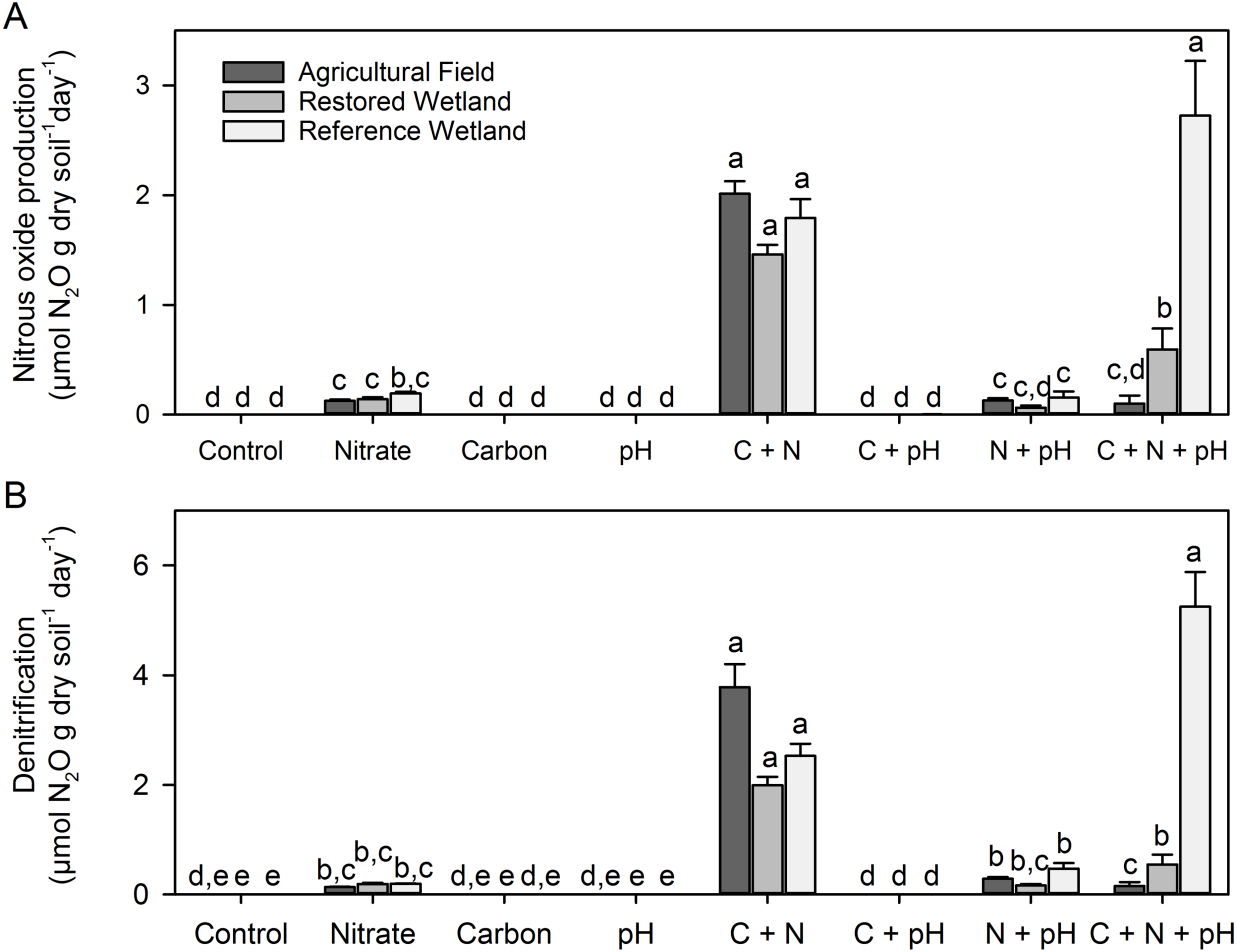
N_2_O production and denitrification rates in laboratory incubations (A) Nitrous oxide production and (B) denitrification rate in all treatments and sites (C, carbon amendment, N, nitrogen amendment) in the laboratory incubation. Error bars represent one standard error from the mean and different small letters denote significant differences among treatments and sites (*p* < 0.05).

Changing salinity (KCl amendment; salt control) had no effect on CO_2_ or CH_4_ production (*p* ≥ 0.47), but did increase mean N_2_O production and denitrification slightly by 0.001 µmoles N_2_O g dry soil^−1^ day^−1^ (*p* ≤ 0.05). Compared to the KNO_3_ treatment, we considered this change negligible (see scale of Fig. 2 and results below).

**Figure 3:**
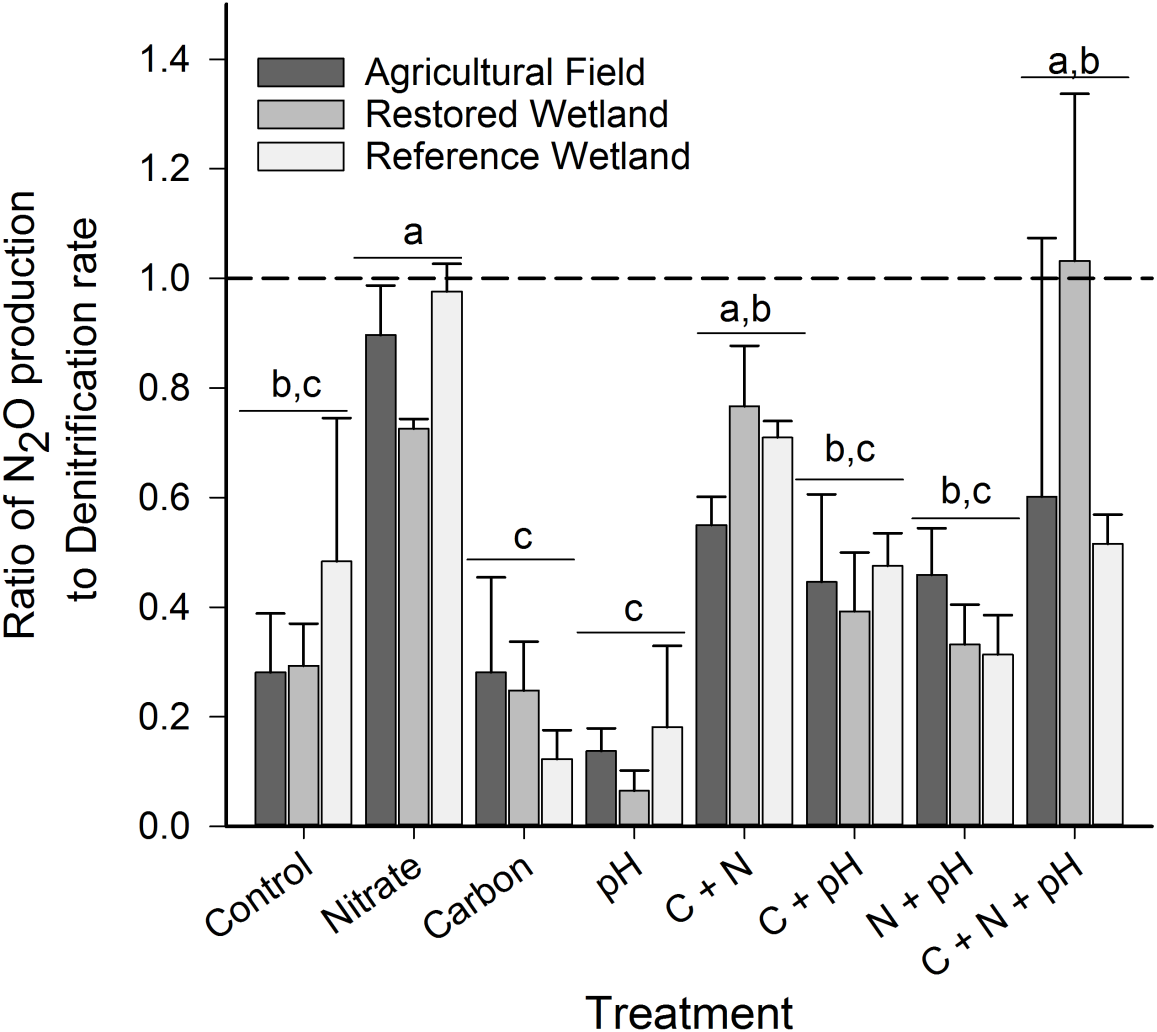
N_2_O production:total denitrification ratio in laboratory incubations. Ratio of N_2_O produced to total denitrification in all treatments and sites in the laboratory incubations. Error bars represent one standard error from the mean and different small letters denote significant differences among treatments across sites (*p* < 0.05).

N_2_O production and denitrification responded similarly to our soil amendments (Table S4, Fig. 2) and were highly correlated (*r*^2^ = 0.93, *p* < 0.001). When soil amendments were added individually (and not in factorial combination), only the addition of nitrate stimulated N_2_O production and denitrification (*p* < 0.05, Fig. 2). However, the factorial combination of nitrate and carbon resulted in an even greater increase in both N_2_O production (10-fold increase over NO_3_^−^ addition alone) and denitrification rates (15-fold increase). We observed no differences among sites except when soils were amended with nitrate and carbon, and pH was altered (C + N + pH treatment, Fig. 2). In this factorial combination, the reference wetland had the highest observed rates, followed by the restored wetland, with the agricultural field having the lowest rates. This was also the only treatment that altering pH had an effect, where neutralizing the pH of the agricultural field and restored wetland decreased N_2_O production and denitrification compared to soils only amended with carbon and nitrate (C + N). The ratio of N_2_O produced to total denitrification (Fig. 3) did not depend on site (*p* ≥ 0.17, Table S4). In general, when nitrate was added to the soils the proportion mineralized as N_2_O increased to near 100%. In the altered pH only and C-amendment only treatments, less than 22% of denitrification was mineralized as N_2_O.

**Figure 4:**
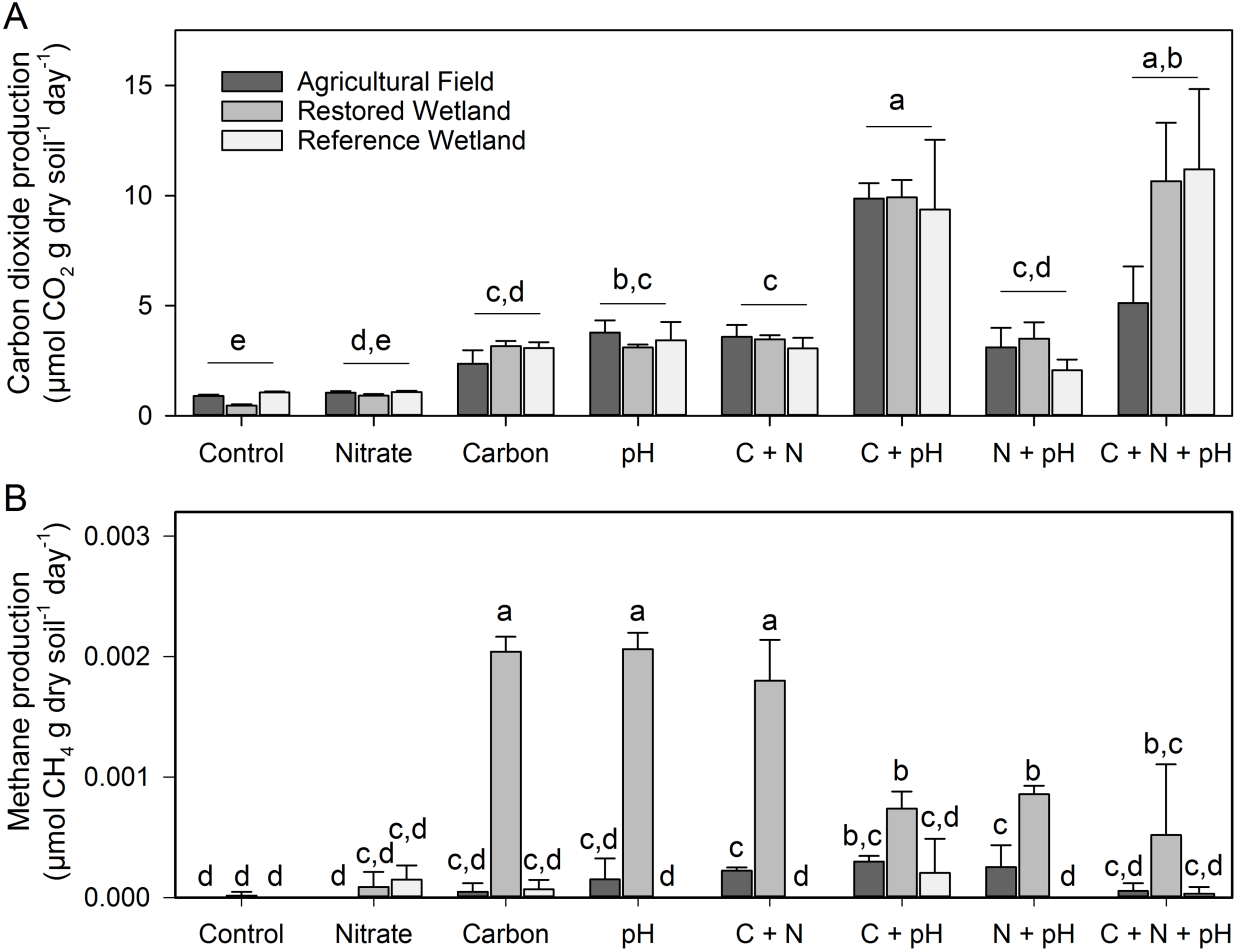
Carbon dioxide and methane production in laboratory incubations. (A) Carbon dioxide and (B) methane production in all treatments and sites in the laboratory incubations (C, carbon amendment, N, nitrogen amendment). Error bars represent one standard error. For carbon dioxide, small letters denote differences among treatments across sites as there was no site by treatment interaction. For methane production, where there was such an interaction, small letters indicate significant differences among treatments and sites (*p* < 0.05).

Carbon dioxide production did not vary with site or nitrate amendment in the laboratory incubation (Table S4, Fig. 4A). However, the carbon amendment and increase in pH stimulated CO_2_ production, and in factorial combination (C + pH) the stimulation was additive (interaction *p* = 0.107, Table S4, Fig. 4) causing a production increase of at least an order of magnitude.

Methane production varied considerably by site and treatment (Site + N + C + pH interaction, *p* < 0.001, Table S4), but overall values were quite low (largest rate measured = 0.002 µmoles CH_4_ g dry soil^−1^ day^−1^, Fig. 4B). As a proportion of total carbon respired, at most only 0.1% was from CH_4_ production. We observed the largest increases in CH_4_ production in the restored wetland, with all treatments being significantly greater than the control except the nitrate-only amendment (Fig. 4B). The agricultural field showed a slight increase in CH_4_ in the C + N, C + pH, and N + pH treatments. The reference treatment did not vary by treatment, with production near zero in all treatments.

## Discussion

We draw three primary conclusions from this work: (1) restoration treatments in short-term may have minimal effects on belowground functioning when soil and hydrology are initially intact; (2) seasonal wetlands of the Willamette Valley, Oregon are not significant sources of the potent greenhouse gases CH_4_ and N_2_O due to low soil nitrogen and carbon availability for N_2_O production and perhaps high amounts of alternative e^−^ acceptors for CH_4_ production, and (3) these wetlands have a near zero-climate forcing effect and thus should have minimal influence on the radiative budget. Below, we discuss each of our major findings.

### Restoration influence on belowground functioning

We found minimal influence of restoration treatments on belowground functioning after one year, despite observing large differences in plant community composition during this time period (Pfeifer-Meister et al., 2012b). However, the restorations did show a rapid divergence from their antecedent agricultural conditions to a more ‘natural’ state, with lower nutrient availability (Fig. S1), soil respiration (Fig. 1A), and net nitrogen mineralization and nitrification rates (Figs. 1B, 1C) in various seasons. Moreover, as part of another study, nutrients were measured in three native remnant wetlands in the spring (Pfeifer-Meister et al., 2012a), and when compared to the restoration treatments in this study during the same time period, NH_4_^+^ and NO_3_^−^ availability did not significantly differ (*p* > 0.30). This was also true when soils were collected for the laboratory experiment that was used to explore controls of trace gas production (Table 1), suggesting that after restoration nutrient levels quickly returned to background levels typical of native wetland prairies. Given that many potential wetland restoration sites throughout the US are currently in agriculture and receive fertilization, this is a promising result. However, available nitrogen concentrations returned to low, background levels in the agricultural field in the winter and spring, and for NH_4_^+^ also in the summer, suggesting a prudent nutrient management regime (Fig. S1). The degree of long-term nutrient enhancement of the soil from past agricultural practices will depend on the specific management regime within a field. Other sites may maintain higher soil nutrient availability for a longer period post-restoration. Site preparation may also have larger effects on belowground function when using more extreme restoration techniques such as topsoil removal (Holzel & Otte, 2003; Pfeifer-Meister et al., 2012a; Tallowin & Smith, 2001) or restoring site hydrology (Tangen, Finocchiaro & Gleason, 2015).

Contrary to our expectations, fluxes of N_2_O and CH_4_ in the agricultural field and in the experimental restoration plots never significantly differed from zero, nor did they in our previous study examining six restored wetlands (ranging one to seven years old) and three native remnant wetlands (Pfeifer-Meister et al., 2012a). Greenhouse gas fluxes are rarely compared among natural, agricultural, and restored wetlands, and of the studies that have compared these fluxes, some found that restored sites had higher greenhouse gas emissions than remnants (Badiou et al., 2011), others found lower rates (Nahlik & Mitsch, 2010), and still others saw no difference between natural and restored sites (Richards & Craft, 2015). Moreover, Tangen, Finocchiaro & Gleason (2015) showed that the response of trace gases to restoration is dependent upon whether restored wetlands were formerly drained or intact. However, all of these studies have come from areas experiencing a temperate continental climate (Canada, Ohio, Indiana, and North Dakota, respectively), and none with a strong Mediterranean climate as in our system. Nitrous oxide emissions are often very high in agricultural fields (Bedard-Haughn, Matson & Pennock, 2006; Liu & Greaver, 2009; Mosier et al., 1991), but the prudent nutrient additions and high cation exchange capacity of the 2:1 clays (Brady & Weil, 2010) appear to have minimized these emissions in our study sites.

### Why are fluxes of N_2_O and CH_4_ zero?

Nitrous oxide is produced in soils primarily through the microbial-mediated processes of nitrification and denitrification, which accounts for an estimated 50% of global N_2_O terrestrial flux into the atmosphere (80% of terrestrial emissions when agriculture is included; Ciais et al., 2013). In nitrification, nitrous oxide can be released as an intermediate product in the conversion of NH${}_{4}^{+}$ to NO${}_{3}^{-}$, where a small proportion of the N is lost through a “hole-in-the-pipe” (Firestone & Davidson, 1989). This is generally an aerobic process that is predominantly controlled by the supply of NH${}_{4}^{+}$ and soil moisture of the system. In general, net nitrification (and mineralization) rates were low in this system (average: 0.04 µg N g^−1^ day^−1^ ±  0.01, range: −0.39 to 1.76, Fig. 1), with the highest rates observed in the agricultural field during the fall and summer season. Average rates in the restored plots were comparable to an upland prairie in the region (Pfeifer-Meister & Bridgham, 2007) where soil moisture was a dominant control. Similarly, in the present study, rates were highest during the warmer fall and summer months when water availability was moderate (*r* =  − 0.18, *p* = 0.006) and were not tightly coupled with available NH_4_^+^ (*r* = 0.004, n.s.) or temperature (*r* = 0.12, n.s.). Given these low nitrification rates, it is unlikely that nitrous oxide would be a significant by-product as, at most, this would be a small proportion of total nitrification (Chapin III, Matson & Mooney, 2002).

Nitrous oxide is also an intermediate in denitrification (the conversion of NO_3_^−^ or NO_2_^−^ to N_2_ under anaerobic conditions), where NO_3_^−^, organic carbon, and oxygen availability (and thus soil moisture) are considered the primary controls (Bedard-Haughn, Matson & Pennock, 2006; Chapin, Matson & Mooney, 2002; Liu & Greaver, 2009; Senbayram et al., 2012). Empirical (Beringer et al., 2013; Gleason et al., 2009) and theoretical (Davidson et al., 2000) studies have shown that maximum N_2_O emissions occur when water-filled pore space (WFPS) ranges from 40–80%. In our study, during three of the four sampling periods WFPS was within this range, indicating that sufficient moisture was available for N_2_O production to occur (Table S3). However, wetland prairies of this region have low soil carbon and nitrate availability (Table 1 and Pfeifer-Meister et al., 2012a). When we amended soils with these substrates, we observed a significant stimulation of both N_2_O production and denitrification (Fig. 2). These processes were highly correlated (*r*^2^ = 0.93, *p* < 0.01) suggesting that increase in N_2_O production was likely attributed to the increase in denitrification and not nitrification (this is not surprising as our anaerobic incubations would have limited nitrification). Nitrogen appeared to be the primary limiting factor, but when coupled with additions of organic carbon, rates increased by another order of magnitude (10–15 times). The proportion emitted as N_2_O (vs. total denitrification) also increased with nitrate addition (Fig. 3), which has been observed in many other systems (e.g. Senbayram et al., 2012). This has been attributed to the inhibitory effect of NO_3_^−^ on N_2_O reduction (Van Cleemput, 1998). This inhibitory effect is often reduced with increasing abundance of carbon (Senbayram et al., 2012), but we did not observe this decrease in the ratio in our experiment. Importantly, we observed no differences among sites with one exception in the C + N + pH treatment. In this treatment, the change in pH decreased N_2_O and denitrification rates in the agricultural field and restored site but not the reference wetland. One possible explanation is that the microbial communities were not acclimated to the change in pH in these sites (the restored and agricultural sites had a lower native pH than the reference wetlands, Table 1). The co-limitation of nitrate and carbon suggests that even when receiving relatively high levels of nitrogen inputs, organic matter availability will continue to constrain N_2_O emissions and denitrification in these wetlands. This inability to denitrify excess nitrate (from fertilizers, livestock waste, stormwater, etc.) has important implications for the quality of groundwater and surrounding waterways. In the Southern Willamette Valley, this is recognized as an ongoing problem (designated as a Groundwater Management Area) with more than 20% of tested sites exceeding 7 mg N L^−1^ (Oregon State University Well Water Program Staff, 2017).

Methane flux is a result of the simultaneous processes of consumption (methanotrophy) and production (methanogenesis). The oxidative consumption of methane is generally highest under aerobic conditions with a drawdown of the water table below the surface or with extensive plant aerenchyma development causing aeration of the rhizosphere. Many of the times that we sampled CH_4_ fluxes, the water table had been at or above the surface for months in the mild winters and springs of the region, leading us to hypothesize that the bottleneck in CH_4_ flux was in the production side and not the oxidation side of the ledger. The dominant controls of CH_4_ production include anaerobic conditions, organic matter quantity and quality, and the availability of more thermodynamically favorable electron acceptors (Bridgham et al., 2013). Theoretically, methanogenesis should be suppressed in the presence of NO_3_^−^ , Fe (III), Mn (III, IV), oxidized humic substances, and SO_4_^2−^, which are more energetically favorable electron acceptors. Moreover, low pH and temperature may present physiological constraints on microbial communities and both have been shown to limit rates of methanogenesis (Turetsky et al., 2014; Ye et al., 2012).

We never observed significant fluxes of CH_4_, even during periods of extensive water-logging of the soil. In our anaerobic laboratory incubations, the addition of a carbon source and an increase in pH also did not substantially increase rates of methanogenesis (Fig. 4B, highest rate observed = 0.002 µmoles CH_4_g dry soil^−1^ day^−1^, though statistically significant, biologically this increase was inconsequential). Thus, we can eliminate the short-term effects of NO_3_^−^ availability, carbon quantity, and pH as potential mechanisms for the lack of CH_4_ production, although it is possible that these factors may be limiting methanogen population size and/or activity and the five day pre-incubation was insufficient for a response. We did not measure Fe (III), Mn (III, IV), SO_4_^2−^, or oxidized humic substance availability in our soils. It is possible that sufficient quantities of these alternative electron acceptors exist allowing other microbial groups to outcompete methanogens even after prolonged water logging. In support of this hypothesis, we did observe an increase in CO_2_ production in response to added carbon, which in consistent with other microbial groups outcompeting methanogens. In three nearby wet prairies of the same soil series (Natroy) and similar pH, iron concentrations ranged from 130-302 ppm and sulfate concentrations ranged from 2.6–8.1 ppm (Finley, 1994). Furthermore, redox values were generally between 150 mV and −100 mV during the winter and spring months which is sufficient to reduce iron (Finley, 1994), supporting our hypothesis that the biological reduction of available soil Fe(III) may suppress methanogenesis in these wetland prairies. The low C and N content of the soils may also inhibit the respiratory activity of the microbial Fe(III) reducers so that a large pool of oxidizable Fe remains in the soil even after prolonged flooding with mild temperatures.

### Radiative forcing of oregon seasonal wetland prairies

Many studies have demonstrated that wetlands are an important driver of global radiative budgets. Wetlands contain approximately one third of the world’s terrestrial soil carbon (Bridgham et al., 2006; Ciais et al., 2013), and methane flux from wetlands has been linked with interannual variation in climate (Bousquet et al., 2006). More recently, the observed increase in atmospheric methane concentrations has been attributed to an increase in wetland emissions (Bousquet et al., 2011; Parmentier et al., 2015), and this is expected to increase with ongoing climate change with the potential for wetlands to produce more total methane than all anthropogenic sources combined (Zhang et al., 2017). There is also increasing concern that nitrogen deposition and fertilization may contribute to a substantial increase in N_2_O emissions from wetlands, offsetting potential gains in carbon sequestration through nutrient enrichment (Liu & Greaver, 2009). However, there are very few studies with data on CH_4_ and N_2_O emissions and soil carbon sequestration in mineral-soil wetlands to evaluate these concerns, especially in those with different land-use practices.

Though we did not measure a complete carbon budget of our system, several lines of evidence indicate that net radiative forcing of these wetland prairies are minimal at best. First, soil carbon did not differ between the remnant, restored or agricultural field (Table 1). Because the wetlands are not anaerobic year-round, organic matter may decompose more readily during the drier periods and not accumulate at an appreciable rate. The high adsorption capacity of the 2:1 clays may also provide substantial physical protection of soil organic matter. In other seasonal wetlands, no differences have been detected in soil organic carbon between restored and agricultural sites (Gleason et al., 2009). However, in other types of freshwater wetlands, soil carbon is often lower in restored sites than in reference wetlands (Hossler et al., 2011; Richards & Craft, 2015), but often these restorations involve restoring site hydrology. In a meta-analysis, Moreno-Mateos et al. (2012) found that soil carbon was still reduced by 50% even 20 years post-restoration, but this was largely attributed to wetlands being drained prior to restoration. However, in this meta-analysis seasonal temperate wetlands were not included to examine differences in soil carbon pools. Second, fluxes of N_2_O and CH_4_ are insignificant in this system (see ‘Discussion’ above). Third, these wetland prairies have minimal surface flow and do not appear to have significant sedimentation rates (L Pfeifer-Meister, per. obs., 2006). Fourth, the restored treatments reached comparable aboveground NPP to the remnant wetlands two years post-restoration (Fig. S3), indicating a very rapid attainment of steady-state biomass. The fertilized agricultural field had higher productivity than the restoration plots. However, most of that biomass is hayed and removed from the site, where it either decomposes relatively rapidly or is used for fodder for cattle or other livestock, resulting in an indirect increase in radiative forcing through CH_4_ emissions and the associated carbon costs of the livestock industry (Eckard, Grainger & De Klein, 2010). The high soil respiration rates in the agricultural field (Fig. 1) and lack of differences in soil carbon (Table 1) indicate that any additional NPP in the agricultural field is not sequestered within the system.

## Conclusions

Restored seasonal wetland prairies in former agricultural fields that had low rates of fertilization and intact hydrology rapidly attained soil ecosystem functioning that was similar to reference wetlands. We found no effect of restoration on either carbon sequestration or greenhouse gas emissions. This neutral result suggests that these types of West Coast prairie wetlands are inappropriate for carbon credit markets, but it is worth noting that as opposed to many restored wetlands, they also have no warming effect on the climate. Despite this lack of significant carbon sequestration, these wetlands are critically endangered ecosystems and contain important biodiversity, including many federally listed species which more than justifies their continued restoration. It would be worthwhile to pursue similar studies in other wetlands with a Mediterranean climate and vertic clays to decouple these two strong drivers of carbon and nutrient dynamics. Our results may be representative of this class of wetland, which to our knowledge has not been previously assessed for management effects on greenhouse gas fluxes.

##  Supplemental Information

10.7717/peerj.5465/supp-1 Table S1 Restoration treatments implemented for the field experiment The original design included ten site preparation treatment combinations and the farm field. However, the summer herbicide application had no detectable effect on soil response variables (*p* > 0.30), so it was lumped with its equivalent counterpart, reducing the total treatment combinations from ten to seven. For full treatment descriptions see Pfeifer-Meister et al. (2012b).

10.7717/peerj.5465/supp-2 Table S2 *P*-values for soil responses to restoration in the field experiment P-values for one-way and repeated-measures ANOVAs for the effect of restoration treatment and season (repeated-measures only) on soil response variables (*n* = 5). Data were collected in the fall 2005, winter 2006, spring 2006, and summer 2006 in all restoration treatments and agricultural field. For aboveground NPP, we also compared data to the reference site which was collected as part of another study in spring 2005 (Pfeifer-Meister et al., 2012a).

10.7717/peerj.5465/supp-3 Table S3 Seasonal soil moisture, soil temperature, and pH averaged across restoration treatments and agricultural field Mean ± SE soil moisture, soil temperature at a 5 cm depth, and pH averaged across restoration treatments and agricultural field (*n* = 55) for the four sampling seasons. Lower case letter differences indicate significant (*p* < 0.05) differences among seasons. Also reported is the conversion of % moisture to water filled pore space (WFPS) based on an average bulk density of 1.16 g cm^−3^ and an assumed particle density of 2.65 g cm^−3^.

10.7717/peerj.5465/supp-4 Table S4 *P*-values from laboratory incubations *F* and *p*-values from 4-Way ANOVA (total *df* = 119) examining the effect of site, nitrogen amendment (N), carbon amendment (C) and pH alteration on greenhouse gas (CH_4_, CO_2_, N_2_O) production, denitrification, the proportion of CH_4_ produced relative to total carbon production, and the proportion of N_2_O produced relative to total denitrification.

10.7717/peerj.5465/supp-5 Figure S1 Seasonal nutrient availability in the restoration treatments and agricultural field (A) Available ammonium, (B) nitrate, and (C) phosphate in fall 2005, winter 2006, spring 2006, and summer 2006 for the restoration treatments and agricultural field. Error bars represent one standard error from the mean and lower case letter differences indicate significant (*p* < 0.05) effects of treatment within a season. Break in available nitrate panel is from 6 to 15 µg N g soil^−1^ and the two asterisks in panel B indicate that the pairwise comparisons between letters b and c are only marginally significant, *p* < 0.08).

10.7717/peerj.5465/supp-6 Figure S2 Microbial biomass C and N across seasons in restored wetlands and agricultural field Microbial biomass carbon (left *y*-axis) and nitrogen (right *y*-axis) averaged across seasons in restoration treatments and agricultural field. Error bars represent one standard error from the mean and lower case letter (carbon) and number (nitrogen) differences indicate significant effects (*p* < 0.05, ∗*p* < 0.10) among treatments. Note 10-fold difference in magnitude of *y*-axes.

10.7717/peerj.5465/supp-7 Figure S3 NPP in restoration treatments, agricultural field, and reference wetland Aboveground net primary productivity (NPP) in restoration treatments, agricultural field, and reference wetland. Aboveground NPP (g m^−2^ yr^−1^) is further portioned into graminoids, forbs, and woody biomass. Error bars represent one standard error from the mean and lower case letter differences indicate significant effects (*p* < 0.05, ∗*p* < 0.10) among treatments. Note: Biomass data in the restoration treatments and agricultural field were collected June of 2006 and biomass data for the reference site was collected in June of 2005 as part of another study (Pfeifer-Meister et al., 2012a).

10.7717/peerj.5465/supp-8 Supplemental Information 1 Soil properties of restored, agricultural, and natural wetlands used for the laboratory experiment Initial nitrate and ammonium availability, total soil nitrogen and carbon, bulk density, pH, % clay, % sand, and % silt of agricultural, restored, and natural wetlands used in the laboratory incubations. Soil cores from a site were homogenized prior to incubations. Data is presented in Table 1.

10.7717/peerj.5465/supp-9 Supplemental Information 2 Raw data from laboratory incubations File includes the CO_2_ production, CH_4_ production, N_2_O production, denitrification rates, pH, % moisture, Initial and final nutrient availability, and the ration of N_2_O production to total denitrification from the laboratory incubations. This data is presented in Figs. 2–4.

10.7717/peerj.5465/supp-10 Supplemental Information 3 Net Primary Productivity in restoration treatments, agricultural field, and reference wetlands Aboveground net primary productivity (NPP) in restoration treatments, agricultural field, and reference wetland. Aboveground NPP is portioned into graminoids, forbs, and woody biomass. Data is shown in Fig. S3.

10.7717/peerj.5465/supp-11 Supplemental Information 4 Soil properties and in situ fluxes measured seasonally in the restoration treatments and agricultural field Bulk Density, C/N ratio, Total N, Total C, % moisture, pH, available ammonium , nitrate, and phosphate, belowground biomass, microbial C, N and P, net N mineralization, net nitrification, and in situ soil respiration from chambers and soil cores collected in the fall 2005, winter 2006, spring 2006, and summer 2006 for the restoration treatments and agricultural field. Data is presented in Fig. 1, Tables S2–S4, and and Figs. S1–S2.

## Additional Information and Declarations

### Data Availability

The following information was supplied regarding data availability: The raw data are provided in the Supplemental Files.
