# Supplementary material for: Greenhouse gas emissions limited by low nitrogen and carbon availability in natural, restored, and agricultural Oregon seasonal wetlands
Source: PeerJ. 2018 Aug 28;6:e5465. doi: 10.7717/peerj.5465 (PMC6118202; doi:10.7717/peerj.5465)
Supplement: Table S1 — The original design included ten site preparation treatment combinations and the farm field. However, the summer herbicide application had no detectable effect on soil response variables (p > 0.30), so it was lumped with its equivalent counterpart, reducing the total treatment combinations from ten to seven. For full treatment descriptions see Pfeifer-Meister et al. (2012b). [file peerj-06-5465-s001.pdf]

Table S1. Restoration treatments implemented for the field experiment. The original design included ten site preparation treatment combinations and the farm field. However, the summer herbicide application had no detectable effect on soil response variables ($p > 0.30$), so it was lumped with its equivalent counterpart, reducing the total treatment combinations from ten to seven. For full treatment descriptions see Pfeifer-Meister et al. (2012b).

<table>
<tr><th></th><th>Original Treatment</th><th></th><th>Collapsed Treatment</th></tr>
<tr><td>1</td><td>Summer Herbicide</td><td>1</td><td>Control: Summer Herbicide</td></tr>
<tr><td>2</td><td>Till</td><td rowspan="2">2</td><td rowspan="2">Till</td></tr>
<tr><td>3</td><td>Till + Summer Herbicide</td></tr>
<tr><td>4</td><td>Summer Herbicide + Thermal</td><td>3</td><td>Thermal</td></tr>
<tr><td>5</td><td>Till + Thermal</td><td rowspan="2">4</td><td rowspan="2">Till + Thermal</td></tr>
<tr><td>6</td><td>Till + Summer Herbicide + Thermal</td></tr>
<tr><td>7</td><td>Summer and Fall Herbicide</td><td>5</td><td>Fall Herbicide</td></tr>
<tr><td>8</td><td>Till + Summer and Fall Herbicide</td><td>6</td><td>Till + Fall Herbicide</td></tr>
<tr><td>9</td><td>Till + Solarization</td><td rowspan="2">7</td><td rowspan="2">Till + Solarization</td></tr>
<tr><td>10</td><td>Till + Summer Herbicide + Solarization</td></tr>
<tr><td>11</td><td>Agricultural Field</td><td>8</td><td>Agricultural Field</td></tr>
</table>
