# Supplementary material for: Greenhouse gas emissions limited by low nitrogen and carbon availability in natural, restored, and agricultural Oregon seasonal wetlands
Source: PeerJ. 2018 Aug 28;6:e5465. doi: 10.7717/peerj.5465 (PMC6118202; doi:10.7717/peerj.5465)
Supplement: Table S2 — P-values for one-way and repeated-measures ANOVAs for the effect of restoration treatment and season (repeated-measures only) on soil response variables (n = 5). Data were collected in the fall 2005, winter 2006, spring 2006, and summer 2006 in all restoration treatments and agricultural field. For aboveground NPP, we also compared data to the reference site which was collected as part of another study in spring 2005 (Pfeifer-Meister et al., 2012a). [file peerj-06-5465-s002.pdf]

Table S2. P-values for one-way and repeated-measures ANOVAs for the effect of treatment and season (repeated-measures only) on soil response variables (n = 5). Data were collected in the fall 2005, winter 2006, spring 2006, and summer 2006 in all restoration treatments and agricultural field. For aboveground NPP, we also compared data to the reference site which was collected as part of another study in spring 2005 (Pfeifer-Meister et al. 2012a).

**One-way ANOVA** (total df = 55)	**Treatment**		
Bulk Density (g cm^{-3})	0.39		
Total Carbon (g C m^{-2})	0.24		
Total Nitrogen (g N m^{-2})	0.22		
Carbon/Nitrogen Ratio	0.25		
Percent Clay	0.99		
Percent Sand	0.98		
Percent Silt	0.89		
Total Aboveground NPP	**<0.001**		
Grass NPP	**<0.001**		
Forb NPP	**<0.001**		
**Repeated-measures ANOVA** (total df = 220)	**Between**	**Within**	
	**Treatment**	**Season**	**Season*Treatment**
Soil Respiration (mmol CO_2 m^{-2} day^{-1})	**<0.001**	**<0.001**	**0.01**
Net Mineralization (μg N g^{-1} soil day^{-1})	**0.006**	**<0.001**	**0.05**
Net Nitrification (μg N g^{-1} soil day^{-1})	**<0.001**	**<0.001**	**<0.001**
Ammonium (μg N g^{-1} soil)	0.611	**<0.001**	**<0.001**
Nitrate (μg N g^{-1} soil)	**<0.001**	**<0.001**	**<0.001**
Phosphate (μg P g^{-1} soil)	0.70	**<0.001**	0.34
Microbial Carbon (μg C g^{-1} soil)	**0.005**	**<0.001**	0.32
Microbial Nitrogen (μg N g^{-1} soil)	**<0.001**	**<0.001**	0.37
Gravimetric Moisture (%)	0.26	**<0.001**	0.34
pH	**0.005**	**<0.001**	0.33

Values in bold are significant at an alpha < 0.05.
