# Supplementary material for: Greenhouse gas emissions limited by low nitrogen and carbon availability in natural, restored, and agricultural Oregon seasonal wetlands
Source: PeerJ. 2018 Aug 28;6:e5465. doi: 10.7717/peerj.5465 (PMC6118202; doi:10.7717/peerj.5465)
Supplement: Table S3 — Mean ± SE soil moisture, soil temperature at a 5 cm depth, and pH averaged across restoration treatments and agricultural field (n = 55) for the four sampling seasons. Lower case letter differences indicate significant (p < 0.05) differences among seasons. Also reported is the conversion of % moisture to water filled pore space (WFPS) based on an average bulk density of 1.16 g cm−3 and an assumed particle density of 2.65 g cm−3. [file peerj-06-5465-s003.pdf]

Table S3. Mean ± SE soil moisture, soil temperature at a 5 cm depth, and pH averaged across restoration treatment and agricultural field (n = 55) for the four sampling seasons. Lower case letter differences indicate significant ($p < 0.05$) differences among seasons. Also reported is the conversion of % moisture to water filled pore space (WFPS) based on an average bulk density of 1.16 g cm^{-3} and an assumed particle density of 2.65 g cm^{-3}.

Season	% Moisture	WFPS	Soil Temperature (°C)	pH
Fall 2005	20.0 ± 0.2c	41.3%	14.4 ± 0.24b	4.93 ± 0.02b
Winter 2006	36.8 ± 0.5a	76.0%	8.8 ± 0.68d	5.09 ± 0.04a
Spring 2006	31.4 ± 0.4b	64.8%	11.3 ± 0.24c	5.15 ± 0.02a
Summer 2006	13.1 ± 0.3d	27.0%	17.5 ± 0.07a	4.99 ± 0.01b
