# Supplementary material for: Greenhouse gas emissions limited by low nitrogen and carbon availability in natural, restored, and agricultural Oregon seasonal wetlands
Source: PeerJ. 2018 Aug 28;6:e5465. doi: 10.7717/peerj.5465 (PMC6118202; doi:10.7717/peerj.5465)
Supplement: Table S4 — F and p-values from 4-Way ANOVA (total df = 119) examining the effect of site, nitrogen amendment (N), carbon amendment (C) and pH alteration on greenhouse gas (CH4, CO2, N2O) production, denitrification, the proportion of CH4 produced relative to total carbon production, and the proportion of N2O produced relative to total denitrification. [file peerj-06-5465-s004.pdf]

Table S4. F and p-values from 4-Way ANOVA (total df = 119) examining the effect of site, nitrogen amendment (N), carbon amendment (C) and pH alteration on greenhouse gas (CH_4, CO_2, N_2O) production, denitrification, the proportion of CH_4 produced relative to total carbon production, and the proportion of N_2O produced relative to total denitrification.

	**CH_4 production (square root transform)**	**CO_2 production (square root transform)**	**N_2O production (square root transform)**	**Denitrification rate (log transform)**	**CH_4 / (CO_2 + CH_4) (log transform)**	**N_2O/ Denitrification (square root transform)**
Site (df = 2)	F = 152.14, **p < 0.001**	F = 0.37, p = 0.692	F = 31.17, **p < 0.001**	F = 5.20, **p = 0.007**	F = 61.69, **p < 0.001**	F = 0.19, p = 0.828
Nitrogen (df = 1)	F = 0.02, p = 0.903	F = 0.20, p = 0.655	F = 1251.93, **p < 0.001**	F = 2015.3, **p < 0.001**	F = 0.19, p = 0.662	F = 41.88, **p < 0.001**
Carbon (df = 1)	F = 14.04, **p < 0.001**	F = 79.58, **p < 0.001**	F = 346.73, **p < 0.001**	F = 64.6, **p < 0.001**	F = 3.76, **p = 0.056**	F = 1.60, p = 0.210
pH (df = 1)	F = 2.92, p = 0.091	F = 77.25, **p < 0.001**	F = 41.67, **p < 0.001**	F = 0.10, p = 0.758	F = 0.86, p = 0.355	F = 4.51, **p = 0.036**
Site × N (df = 2)	F = 0.11, p = 0.898	F = 0.61, p = 0.547	F = 30.81, **p < 0.001**	F = 10.74, **p < 0.001**	F = 1.44, p = 0.242	F = 0.62, p = 0.541
Site × C (df = 2)	F = 0.79, p = 0.457	F = 1.23, p = 0.298	F = 21.10, **p < 0.001**	F = 7.77, **p = 0.001**	F = 0.25, p = 0.777	F = 1.79, p = 0.173
Site × pH (df = 2)	F = 3.04, **p = 0.053**	F = 0.622, p = 0.539	F = 27.13, **p < 0.001**	F = 5.55, **p = 0.005**	F = 3.46, **p = 0.036**	F = 0.01, p = 0.992
N × C (df = 1)	F = 12.40, **p < 0.001**	F = 0.01, p = 0.926	F = 329.37, **p < 0.001**	F = 5.30, **p = 0.024**	F = 9.98, **p = 0.002**	F = 1.00, p = 0.321
N × pH (df = 1)	F = 25.74, **p < 0.001**	F = 2.83, p = 0.096	F = 46.13, **p < 0.001**	F = 19.15, **p < 0.001**	F = 9.44, **p = 0.003**	F = 4.73, **p = 0.032**
C × pH (df = 1)	F = 31.41, **p < 0.001**	F = 2.65, p = 0.107	F = 20.96, **p < 0.001**	F = 0.84, p = 0.363	F = 32.11, **p < 0.001**	F = 13.39, **p < 0.001**
Site × N × C (df = 2)	F = 1.28, p = 0.284	F = 0.71, p = 0.496	F = 19.87, **p < 0.001**	F = 0.67, p = 0.512	F = 3.73, **p = 0.028**	F = 0.99, p = 0.376
Site × N × pH (df = 2)	F = 5.12, **p = 0.008**	F = 1.39, p = 0.254	F = 26.47, **p < 0.001**	F = 6.04, **p = 0.003**	F = 0.58, p = 0.563	F = 1.30, p = 0.828
Site × C × pH (df = 2)	F = 37.92, **p < 0.001**	F = 0.46, p = 0.634	F = 32.66, **p < 0.001**	F = 5.55, **p = 0.005**	F = 39.36, **p < 0.001**	F = 0.84, p = 0.434
N × C × pH (df = 1)	F = 1.71, p = 0.194	F = 0.05, p = 0.827	F = 26.35, **p < 0.001**	F = 35.24, **p < 0.001**	F = 0.15, p = 0.699	F = 1.41, p = 0.238
Site × N × C × pH (df = 2)	F = 12.28, **p < 0.001**	F = 1.57, p = 0.214	F = 31.99, **p < 0.001**	F = 5.73, **p = 0.004**	F = 6.47, **p = 0.002**	F = 0.20, p = 0.818

Values in bold are significant at an alpha < 0.05.
