# Supplementary figures and images for: Greenhouse gas emissions limited by low nitrogen and carbon availability in natural, restored, and agricultural Oregon seasonal wetlands
Source: PeerJ. 2018 Aug 28;6:e5465. doi: 10.7717/peerj.5465 (PMC6118202; doi:10.7717/peerj.5465)

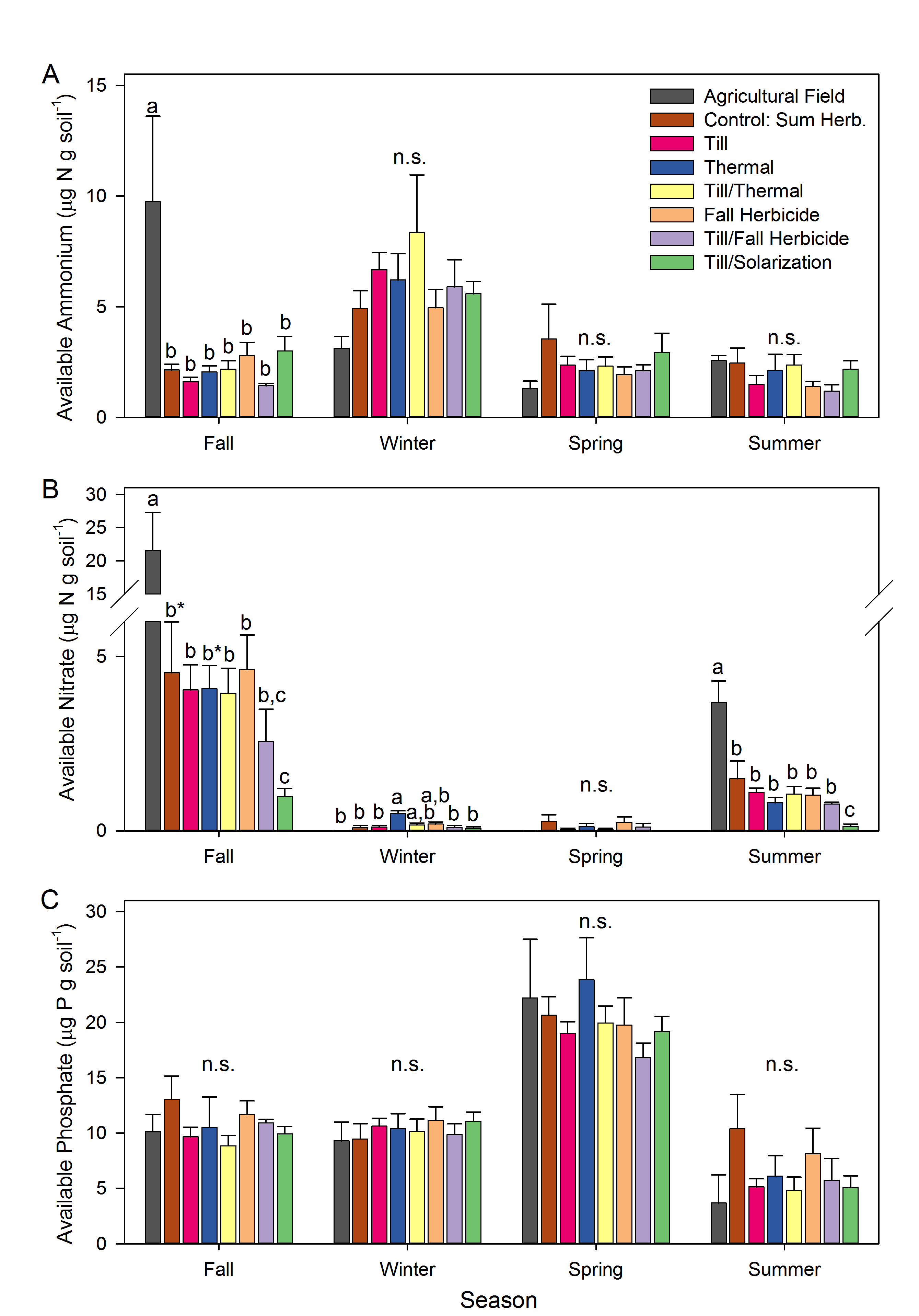

Supplement: Figure S1 — (A) Available ammonium, (B) nitrate, and (C) phosphate in fall 2005, winter 2006, spring 2006, and summer 2006 for the restoration treatments and agricultural field. Error bars represent one standard error from the mean and lower case letter differences indicate significant (p < 0.05) effects of treatment within a season. Break in available nitrate panel is from 6 to 15 µg N g soil−1 and the two asterisks in panel B indicate that the pairwise comparisons between letters b and c are only marginally significant, p < 0.08). [file peerj-06-5465-s005.png]

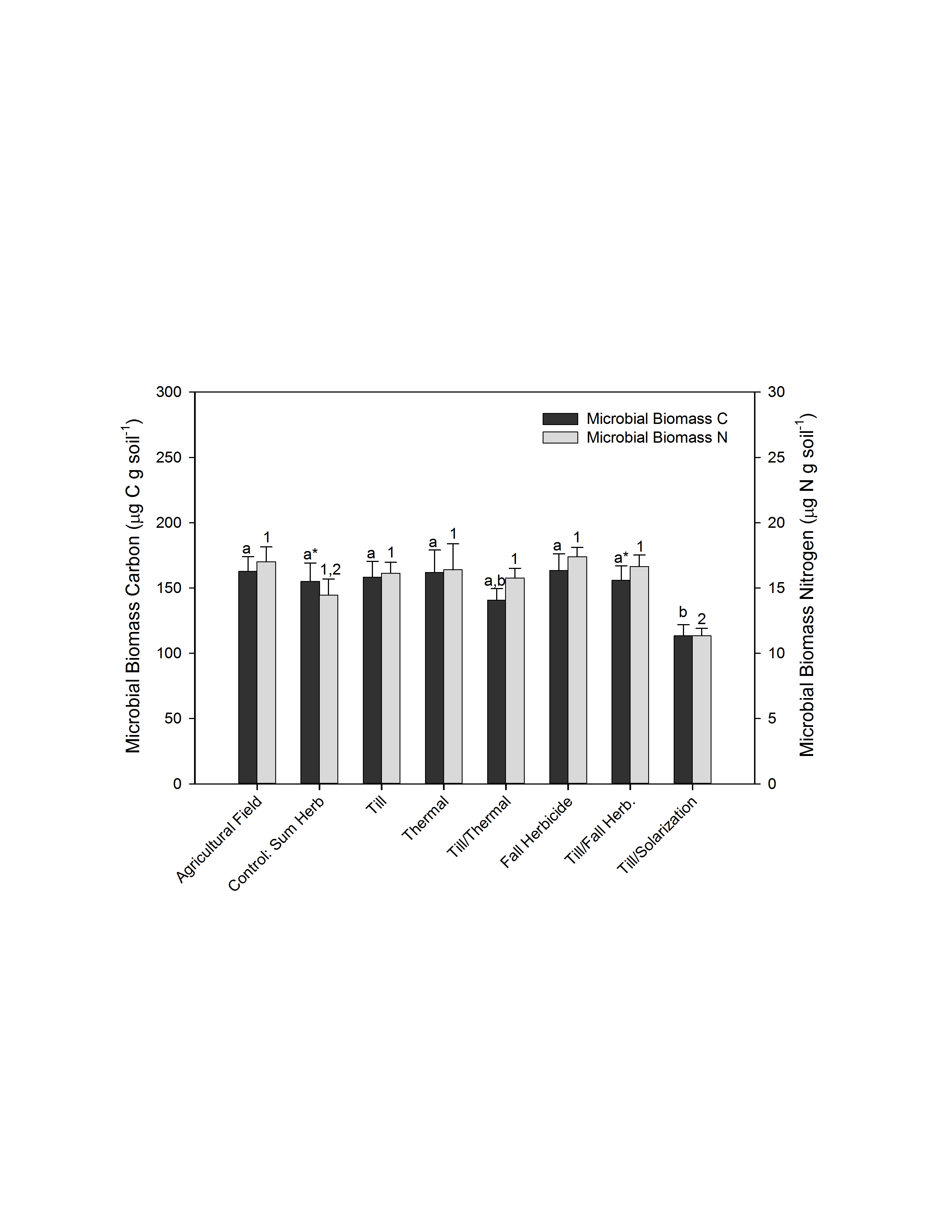

Supplement: Figure S2 — Microbial biomass carbon (left y-axis) and nitrogen (right y-axis) averaged across seasons in restoration treatments and agricultural field. Error bars represent one standard error from the mean and lower case letter (carbon) and number (nitrogen) differences indicate significant effects (p < 0.05, ∗p < 0.10) among treatments. Note 10-fold difference in magnitude of y-axes. [file peerj-06-5465-s006.png]

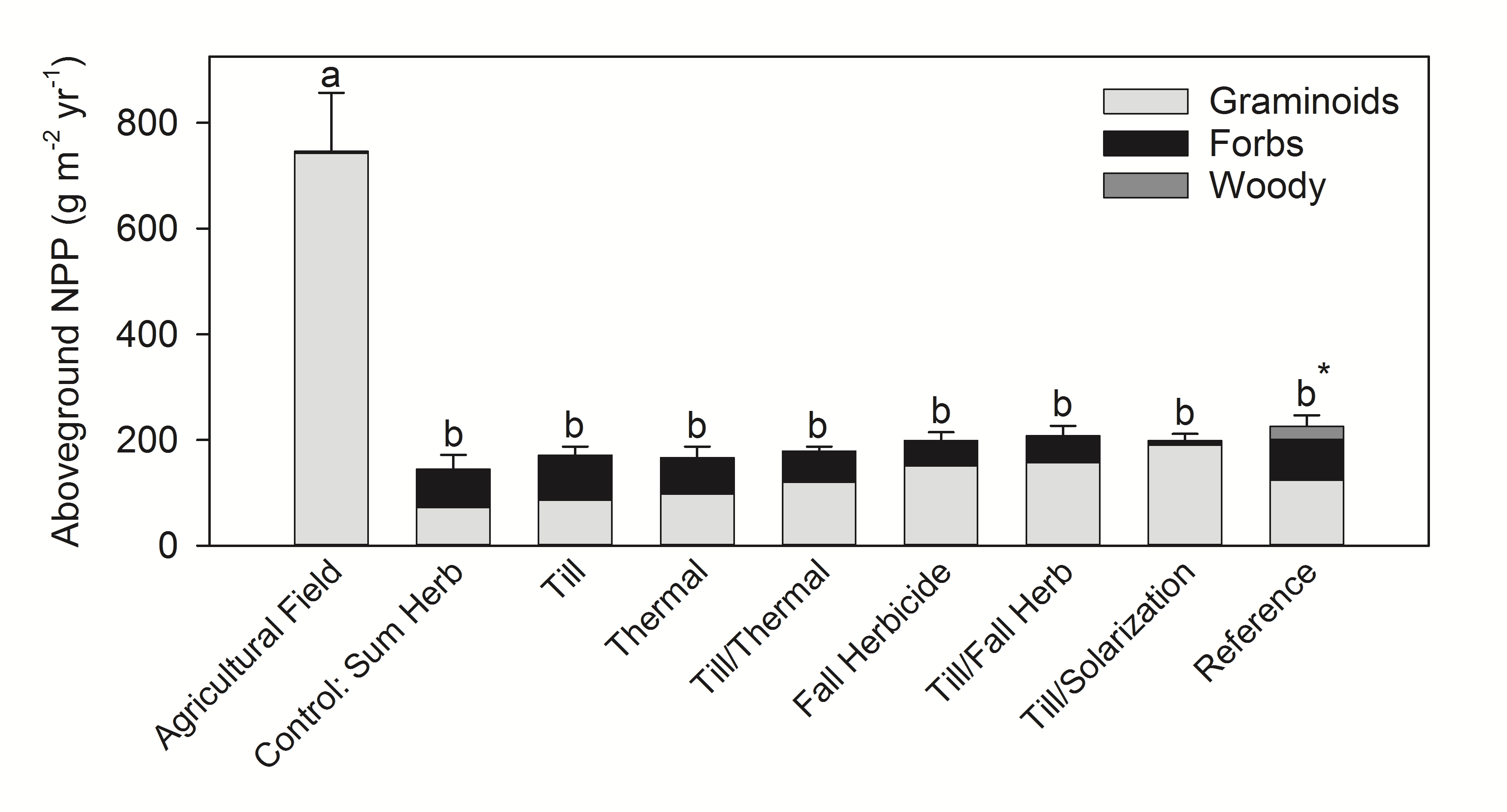

Supplement: Figure S3 — Aboveground net primary productivity (NPP) in restoration treatments, agricultural field, and reference wetland. Aboveground NPP (g m−2 yr−1) is further portioned into graminoids, forbs, and woody biomass. Error bars represent one standard error from the mean and lower case letter differences indicate significant effects (p < 0.05, ∗p < 0.10) among treatments. Note: Biomass data in the restoration treatments and agricultural field were collected June of 2006 and biomass data for the reference site was collected in June of 2005 as part of another study (Pfeifer-Meister et al., 2012a). [file peerj-06-5465-s007.png]
